# Results of 15 years of extended follow-up of the German porcelain workers cohort study: lung cancer and silicosis

**DOI:** 10.3389/fpubh.2025.1552687

**Published:** 2025-03-18

**Authors:** Thomas Birk, Kenneth A. Mundt, Lori Crawford, Patrizia Driesel

**Affiliations:** ^1^Exposure Assessment, Risk Assessment & Risk Management, Bochum, Germany; ^2^Department of Biostatistics and Epidemiology, School of Public Health and Health Sciences, University of Massachusetts Amherst, Amherst, MA, United States; ^3^Health Sciences, Ramboll, Arlington, VA, United States; ^4^Verwaltungs-Berufsgenossenschaft (VBG), Würzburg, Germany

**Keywords:** respirable crystalline silica, silicosis, lung cancer, occupational epidemiology, exposure thresholds

## Abstract

**Objectives:**

To quantify, after extending follow-up 15 years, the relationship between occupational respirable crystalline silica (RCS) exposure and risk of silicosis diagnosis and lung cancer mortality in the German Porcelain Workers Cohort Study, and to estimate possible exposure thresholds for these.

**Methods:**

Porcelain workers enrolled between January 1, 1985, and December 31, 1987, in a mandatory medical surveillance program including triennial chest x-rays and alive at the end of the previous study follow-up (2005) were followed through December 2020, for lung cancer mortality and silicosis incidence. Cause of death was determined from death certificates. Silicosis cases were identified by re-reading x-rays of individuals remaining in the medical surveillance program or filing insurance claims for silicosis. RCS exposure was estimated for each cohort member using a job exposure matrix (JEM) based on about 8,000 historical industrial hygiene RCS measurements. Cause-specific standardized mortality ratios (SMRs) and Cox proportional hazards ratios (HRs) and their 95% confidence intervals (95% CIs) were estimated by cumulative and average exposure groups, controlling for age, sex, smoking status and employment duration. Exposure-response analyses were performed to identify possible exposure thresholds for lung cancer and silicosis risk.

**Results:**

Total deaths increased from 1,610 (9.1%) to 4,586 (26%) over 537,129 total person-years at risk. All-cause mortality was elevated among men (SMR = 1.10, 95% CI 1.06–1.14); however, a deficit was seen among women (SMR = 0.93, 95% CI 0.89–0.98). No statistically significantly increased mortality was seen due to lung cancer, renal cancer, or non-malignant renal disease – conditions reportedly associated with RCS exposure. Lung cancer mortality was unrelated to RCS exposure level. However, for silicosis cases classified using International Labor Organization (ILO) categories ≥1/1 or 1/0, risk was strongly associated with estimated average exposure >0.10 mg/m^3^ and 0.15 mg/m^3^, and cumulative exposure >3.0 mg/m^3^-years and > 1.0 mg/m^3^-years, respectively.

**Conclusion:**

Despite the large number (*n* = 284) of lung cancer deaths and high historical RCS exposures, no excess risk and no relationship with exposure level were seen. However, RCS exposure was strongly associated with silicosis risk, with clear exposure thresholds. This study further confirms the lack of increased lung cancer risk at RCS levels historically prevalent in the German porcelain industry and that exposures exceeding estimated thresholds clearly increased silicosis risk. Occupational exposure levels in the German porcelain industry in recent decades have remained well below these thresholds; therefore, few additional silicosis cases are expected.

## Introduction

The causal relationship between exposure to respirable crystalline silica (RCS) and risk of silicosis is well established, although the association between RCS exposure and risk of lung cancer is less clear. Furthermore, the exposure thresholds at which RCS substantially increases silicosis risk—and possibly lung cancer risk—is substantially increased has not been established. The latest systematic review of the epidemiological studies in which exposure to RCS was quantitatively estimated and silicosis was diagnosed based on chest radiographs reported that all nine higher-quality studies reported large and statistically significant relative risks; in contrast, some but not most higher-quality epidemiological studies reported statistically significantly increased risks of lung cancer mortality (1). IARC Monograph 68 concluded: “There is sufficient evidence in humans for the carcinogenicity of inhaled crystalline silica in the form of quartz or cristobalite from occupational sources,” additionally noting “… that carcinogenicity in humans was not detected in all industrial circumstances studied. Carcinogenicity may be dependent on inherent characteristics of the crystalline silica or on external factors affecting its biological activity or distribution of its polymorphs” (2) (p. 215). In the most recent IARC review, the Working Group (meeting in 2009) reiterated the classification of crystalline silica as a Group 1 or “known” human carcinogen and again pointed out that the association was not seen in all studies or industrial settings, and relied on pooled studies [such as (3)] and meta-analyses (reporting the largest RRs among those with silicosis and few lung cancer deaths among those without prior silicosis) to support their conclusion (4).

Since the last IARC review, Ge et al. (5) published results from the largest pooled case–control study conducted in Europe and Canada, reporting among many positive associations an odds ratio of 1.15 (95% CI 1.04–1.27) for lung cancer at very low RCS exposure levels (exposure category >0—<0.39 mg/m^3^-years). On the other hand, the original German Porcelain Workers Cohort Study observed no association with lung cancer—even at cumulative exposures 10 times greater. That study, however, reported a clear non-linear (i.e., threshold) relationship between RCS exposure and risk of silicosis graded as 1/1 or higher based on the International Labor Organization (ILO) classification (6, 7). The US OSHA Final Rule on Respirable Crystalline Silica considered these negative lung cancer findings in their ruling, but suggested that the lack of increased risk of lung cancer might not reflect the absence of an effect but rather an inadequate latent period, given the relatively short median follow-up of about 15 years (8).

To shed additional light on the RCS exposure and risk of lung cancer and silicosis, we report here updated results based on 15 years of additional follow-up of the German Porcelain Workers Cohort Study, extending follow-up through December 31, 2020. We also report on the exposure-response relationships between quantitative estimates of RCS exposure and silicosis incidence, including the larger group of silicosis cases graded as 1/0 on the ILO scale, as well as lung cancer deaths.

## Methods

### Original cohort study

The original German Porcelain Workers Cohort Study population, cohort definition, methods used to identify deaths and silicosis morbidity, and methods to estimate individual exposure to RCS were described previously (6, 7, 9). Briefly, we identified 20,039 workers employed at more than 100 plants of the German porcelain industry who participated in the preventive medical surveillance program for early identification of radiological signs of silicosis between January 1, 1985, and December 31, 1987. Many of these workers experienced high historical RCS exposures, with few other workplace lung cancer hazards, e.g., asbestos, hexavalent chromium, or radon and diesel fume as reported in studies of underground miners, limiting the potential for confounding in the porcelain manufacturing setting. Information on smoking also was available for a large proportion of the cohort.

Based on paper employment records for approximately 18,000 workers, and excluding individuals employed less than six cumulative months, 17,644 workers formed the original cohort. Follow-up through 2005 generated over 338,000 person-years at risk and identified 1,595 total deaths (9.2% of the cohort), 94 due to lung cancer, but only 5 deaths due to silicosis (6). All chest radiographs for cohort members with at least one chest radiograph evaluated as 1/0 or higher based on the International Labor Organization (ILO) classification scheme in the preventive medical surveillance program were re-read by specially trained teams of two radiologists certified in the B-reading and classification of chest radiographs for pneumoconiosis according to ILO 2000, blinded to the original ratings of the health insurance. Where re-readings disagreed, a third reader was involved to adjudicate any remaining differences. This process identified and statistically analyzed 40 incident cases of silicosis with ILO score ≥ 1/1 (7).

RCS concentrations were estimated based on statistical modeling of over 8,000 industrial hygiene measurements from approximately 100 production area/job task code combinations and standardized as milligrams of silica per cubic meter of air (mg/m^3^) respirable mass as an 8-h time-weighted average (TWA) and summarized into six primary similar exposure groups (SEGs). Values for each SEG were summed into 5-year periods to form a Job Exposure Matrix (JEM). Detailed work history records were linked with JEM average annual exposure concentrations for each job held and summed to derive cumulative exposure estimates (9).

A formal threshold analysis based on the original study data was performed using Cox regression with restricted cubic splines and fractional polynomials. An average exposure threshold of 0.25 mg/m^3^ (95% CI 0.15–0.30) was estimated for silicosis incidence (ILO ≥1/1) but none could be derived for cumulative exposure (10).

### Cohort study update

For the study update, the approximately 15,000 cohort members alive (and not lost to follow-up) at the end of the first follow-up (i.e., December 31, 2005) were followed for cause-specific mortality and silicosis incidence through Dec 31, 2020, applying the same methods used in the original study. Key methodological features are summarized below.

#### Cause of death and silicosis incidence determination

Vital status for the update was determined based on information from the central population registry for Bavaria (ZEMA) and from responses to written enquiries to community registration offices for residential histories and last known residence. The underlying cause of death for each decedent was obtained from the official death certificate obtained from the community health department in the town or city where the death occurred. Underlying cause of death was coded by a professional nosologist according to the 10th revision of the International Classification of Diseases (ICD-10).

To identify incident silicosis cases, all radiographs performed on the study cohort or reported to the statutory health insurance program (i.e., Workers Compensation) since January 1, 2006, were obtained from the preventive medical surveillance program and re-read as in the original study. Because the routine medical screening program was made voluntary for 2 years (i.e., 2012–2013) we were unable to identify silicosis cases among individuals that discontinued participation. To evaluate the influence of silicosis definition on the risk estimates we additionally analyzed silicosis cases classified based on chest radiographs classified as having an ILO score of 1/0, the level US OSHA and MSHA consider diagnostic for silicosis.

### Exposure information

Because RCS exposure in the porcelain industry since the mid-1970s remained uniformly low—and in most departments negligible—we did not update the previous job exposure matrix or individual quantitative exposure assessment (9).

### Statistical methods

For consistency, and to allow observing changes over time, we performed the same statistical analyses as in the original study using Stata (StataCorp. 2011. Stata Statistical Software: Release 12. College Station, TX: StataCorp LLC). Briefly, we calculated basic standardized mortality ratios (SMRs) for 24 major cause of death categories and used Cox proportional hazards models to evaluate the relationships between individual, time-dependent cumulative and average exposure estimates and (a) silicosis morbidity and (b) lung cancer mortality. Because follow-up time differs for mortality and silicosis morbidity outcomes, separate estimates of person-time were generated. For the SMR analysis, person-time accrued until death, or end of follow-up. For silicosis morbidity, the date of the first x-ray meeting the silicosis definition (i.e., ≥1/0 or higher on the ILO scale) was recorded as the date of diagnosis. Person-time for each cohort member was accumulated until the later of the date of the last available x-ray or the date of silicosis diagnosis. We used age at end of follow-up as the time scale variable in all Cox models, and additional variables considered included sex, smoking status (i.e., ever, never and unknown), age at hire, and duration of employment. For comparability, we used the same cumulative exposure cut points as the previous analysis (7).

## Results

As of the end of follow-up (December 31, 2020), we ascertained vital status for 93.2% of the men and 90.6% of the women (91.9% combined). Person-years (p-y) at risk increased from 338,495 to 537,129, slightly more than half accrued by women. About one third of men were deceased (33.9%) whereas less than one fifth of women had died (19.0%), or about 26% overall (see Table 1). Known smokers represented about 38% and known non-smokers about 31% of the cohort, based on medical records, and the balance had unknown smoking status. Roughly one-fourth of the cohort fell into each of four decades of employment groups. The proportion of cohort members with unknown vital status at the end of the follow-up (1,436) increased from the original study, slightly more among women. Assuming cohort members lost to follow-up died in the same proportion as those successfully traced, we likely missed approximately 190 and 166 deaths among men and women, respectively.

**Table 1:** Vital status category by sex for original study and 15-year update.

	Men						Women					
	Original study			Update			Original study			Update		
	n	%	Person-years	n	%	Person-years	n	%	Person-years	n	%	Person-years
Total	8,288	100.0	156,713	8,289	100.0	243,640.2	9,356	100.0	181,782	9,353	100.0	293,489.3
Alive	6,707	80.9		4,920	59.4		8,228	87.9		6,700	71.6	
Deceased	1,126	13.6		2,808	33.9		484	5.2		1,778	19.0	
Unknown	455	5.5		561	6.8		644	6.9		875	9.4	

Of the 4,586 total observed deaths (26% of the original cohort), 2,808 occurred among men, 873 (31.1%) due to cancers and 925 (32.9%) due to circulatory system diseases. Of the total cancer deaths among men, 194 (22.2%) were due to lung cancer (types not specified). Among the 1,778 female decedents, 557 (31.3%) were due to cancers and 607 (34.1%) due to circulatory system diseases. Of the total cancer deaths among women, 90 (16.2%) were due to lung cancer.

### Standardized mortality ratios for specific cause-of-death categories

SMRs were calculated for 24 major cause-of-death categories for the entire cohort stratified by sex and using the German population as the referent. Supplementary Tables 1, 2 present SMRs and 95% confidence intervals (CI) for each cause-of-death category both based on the original follow-up through 2005 and on the updated follow-up through 2020. Although not among our study hypotheses, SMR results for all analyzed specific cause-of-death categories are reported for completeness and transparency. SMRs also were calculated for the Bavarian sub-cohort, which constituted the majority of the full cohort (84 and 86.0% of total men and women, respectively). Because of substantial overlap, these results were similar to those of the full cohort and are not presented (available upon request).

### Standardized mortality ratios for specific cause-of-death categories of *a priori* interest

We observed no statistically significantly increased risk of mortality due to any of the other causes of death of *a priori* interest based on results of other published studies of silica-exposed workers, i.e., lung and kidney cancers, other diseases of the respiratory system and renal disease (see Table 2). Among men, about 20 fewer lung cancer deaths were observed than expected, and among women, the number observed was essentially that expected.

**Table 2:** Summary SMR results for all deaths, all cancers, and specific causes of death of *a priori* interest by sex.

		Men		Women	
Cause of death	ICD-10	Observed	SMR (95% CI)	Observed	SMR (95% CI)
All causes	A00–Y98	2,808	1.10 (1.06–1.14)	1,778	0.93 (0.89–0.98)
Malignant neoplasms	C00–C97	873	1.09 (1.02–1.17)	557	0.87 (0.80–0.95)
Lung cancer	C34	194	0.90 (0.78–1.04)	90	0.98 (0.80–1.21)
Kidney cancer	C64	23	1.01 (0.67–1.52)	14	1.11 (0.66–1.88)
Diseases of the respiratory system	J00–J98	178	1.06 (0.92–1.23)	114	1.03 (0.86–1.24)
Renal disease	N00–N08, N10-N12, N14–N19, N26–N29	22	0.71 (0.47–1.08)	18	0.61 (0.38–0.97)

Despite the continued lack of evidence of any excess risk of lung cancers among men or women, we further evaluated lung cancer risks by quantitative exposure categories including quantified estimates of cumulative and overall average exposure, as well as duration of employment (see Table 3).

**Table 3:** Lung cancer hazard ratios (HRs) and 95% confidence intervals (95% CI) for men and women by categories of cumulative exposure (mg/m^3^-years), average exposure (mg/m^3^), and duration of employment (decades), all controlling for smoking; and smoking status.

	Men		Women	
	Observed	HR (95% CI)	Observed	HR (95% CI)
Cumulative exposure				
≤0.5	45	Reference	27	Reference
>0.5–1.0	25	0.8 (0.5–1.3)	22	1.0 (0.6–1.8)
>1.0–1.5	18	0.8 (0.5–1.4)	7	0.5 (0.2–1.2)
>1.5–3.0	35	0.9 (0.6–1.5)	16	0.7 (0.4–1.4)
>3–4	13	1.2 (0.7–2.3)	8	1.1 (0.5–2.6)
>4–5	10	1.2 (0.6–2.4)	4	0.8 (0.3–2.2)
>5–6	14	1.5 (0.8–2.8)	2	0.5 (0.1–2.0)
>6	34	1.0 (0.6–1.6)	4	0.6 (0.2–1.7)
Average exposure^‡^				
≤0.05	85	Reference	58	Reference
>0.05–0.1	44	1.5 (1.0–2.3)	13	0.4 (0.2–0.9)
>0.1–0.15	15	1.2 (0.7–2.1)	13	0.9 (0.4–1.7)
>0.15–0.2	28	2.2 (1.4–3.6)	5	0.5 (0.2–1.3)
>0.2	22	1.3 (0.8–2.2)	1	0.4 (0.1–2.7)
Years employed				
≤10	25	Reference	14	Reference
>10–20	36	0.7 (0.4–1.2)	23	0.7 (0.4–1.5)
>20–30	42	0.7 (0.4–1.1)	25	0.7 (0.4–1.3)
>30	91	0.7 (0.5–1.2)	28	0.7 (0.4–1.4)
Smoking				
Never	5	Reference	16	Reference
Ever	120	17.9 (7.3–43.7)	44	6.1 (3.4–10.9)
Unknown	69	7.5 (3.0–18.6)	30	1.6 (0.9–2.9)

Age was used as the time variable. ^‡^Additionally adjusted for duration of employment.

Relative risk estimates did not increase with increasing level of any the three exposure indicators. Only one specific exposure category (i.e., average exposure >0.15–0.2 mg/m^3^ among men) produced a statistically significantly elevated HR; however, the same exposure category for women did not indicate an increased risk, nor was higher average exposure related to lung cancer risk in either men or women.

Risks clearly were high, however, among the subset known to be smokers (HR = 17.9; 95% CI 7.3–43.7 and HR = 6.1; 95% CI 3.4–10.9) for men and women, respectively, compared with known never smokers. Those with unknown smoking status generated intermediate relative risk estimates. Analyses of lung cancer mortality by RCS exposure category and stratified by smoking status generated similar results (results not shown) to the models adjusting for smoking. HRs were similarly null, but slightly underestimated and slightly overestimated in models assuming those with unknown smoking status were smokers and non-smokers, respectively (results not shown), compared with the statistically adjusted model.

### RCS exposure and silicosis defined by ILO score of ≥1/1

In the original study, 40 cohort members (men and women) were classified as having incident or new silicosis diagnoses based on radiographic evidence consistent with ILO score ≥ 1/1. Only six of these cases were diagnosed among women; however, four of these cases were in the two highest cumulative exposure categories (>5 mg/m^3^-years) and likely related to their employment in the porcelain industry. Therefore, all subsequent analyses combined men and women and statistically controlled for sex. During the extended follow-up period, only eight additional silicosis cases (defined as ≥1/1) were verified upon re-reading all available radiographs, resulting in a total of 48 cases for the updated statistical analysis (see Table 4).

**Table 4:** Silicosis (defined as ILO ≥1/1) hazard ratios (HRs) and 95% confidence intervals (95% CI) for the full cohort, lagged 10 years and limited to those hired since 1960, by categories of cumulative exposure (mg/m^3^-years), average exposure (mg/m^3^), and duration of employment (decades), all controlling for smoking; and smoking status.

	HR (95% CI)					
	Full Cohort		Exposure lagged 10 yrs		Limited to those hired since 1960	
	Observed		Observed		Observed	
Cumulative exposure						
≤0.5	4	Reference	5	Reference	4	Reference
>0.5–1.0	2	0.9 (0.1–5.5)	3	1.8 (0.4–8.8)	2	0.9 (0.1–5.3)
>1.0–1.5	2	1.3 (0.2–8.1)	1	0.9 (0.1–8.3)	2	1.3 (0.2–8.4)
>1.5–3.0	2	0.8 (0.1–5.1)	2	1.0 (0.2–6.0)	1	0.8 (0.1–8.3)
>3.0–4.0	3	3.3 (0.6–18.1)	5	7.0 (1.6–30.7)	3	6.1 (1.1–32.8)
>4–5	4	6.5 (1.3–32.4)	4	7.2 (1.5–33.8)	2	10.2 (1.6–66.3)
>5–6	7	10.5 (2.4–46.2)	5	9.6 (2.2–42.5)	1	9.1 (0.9–94.5)
>6	24	9.2 (2.3–36.8)	23	11.3 (3.1–41.8)	5	14.5 (3.0–69.6)
Average exposure^‡^						
≤0.05	6	Reference				
>0.05–0.1	2	0.9 (0.2–4.7)				
>0.1–0.15	4	4.0 (1.1–15.2)				
>0.15–0.2	10	11.1 (3.6–34.4)				
>0.2	26	20.0 (7.5–53.1)				
Years employed						
≤10	3	Reference			3	Reference
>10–20	6	0.8 (0.2–3.5)			6	0.8 (0.2–3.5)
>20–30	15	1.7 (0.4–7.0)			10	1.2 (0.3–5.1)
>30	24	1.2 (0.3–4.7)			1	0.2 (<0.1–2.6)
Smoking						
Never	12	Reference			3	Reference
Ever	24	2.4 (1.2–4.9)			4	4.4 (1.3–15.6)
Unknown	12	0.6 (0.3–1.4)			13	1.6 (0.4–7.1)

Age was used as the time variable. ^‡^Additionally adjusted for duration of employment.

Nearly 80% of silicosis cases defined as ILO score ≥ 1/1 had individual cumulative exposure estimates greater than 3.0 mg/m^3^-years, above which risks were greatly increased for the full cohort as well as those with exposure lagged 10 years and/or hired since 1960, although the number of silicosis cases in the latter group was quite small. Relative risk estimates by average exposure category increased sharply above 0.1 mg/m^3^ but were nearly unrelated to duration of exposure (in decade increments).

Interestingly, and although not generally considered a risk factor for silicosis, smokers were at statistically significantly increased risk.

### RCS exposure and silicosis defined by ILO score of 1/0

Although not evaluated in the original study, we identified a total of 108 cohort members with radiographic evidence of silicosis defined as having an ILO score 1/0 (the level that satisfies the regulatory definition of silicosis in many countries including the United States) based on the radiograph re-reading procedures described above and in greater detail in Mundt et al. (7). These 1/0 cases generally were similar to those with ILO scores ≥1/1 but were slightly more likely to be women and less likely to have had probable RCS exposure prior to their employment in porcelain production (see Supplementary Table 3).

Table 5 presents results for cohort members with silicosis defined as ILO score 1/0, comparable to those presented in Table 4 for cohort members with silicosis defined as ILO score ≥ 1/1.

**Table 5:** Silicosis (defined as ILO 1/0) hazard ratios (HRs) and 95% confidence intervals (95% CI) for the full cohort, lagged 10 years and limited to those hired since 1960, by categories of cumulative (mg/m^3^-years) and average exposure (mg/m^3^), and duration of employment (decades), stratified by sex and controlling for age and smoking.

	HR (95% CI)					
	Full cohort		Exposure lagged 10 years		Limited to those hired since 1960	
	Observed		Observed		Observed	
Cumulative exposure						
≤0.5	6	Reference	9	Reference	6	Reference
>0.5–1.0	7	1.6 (0.5–5.0)	12	3.4 (1.4–8.8)	7	1.8 (0.6–5.4)
>1.0–1.5	13	4.8 (1.7–13.5)	9	4.0 (1.4–10.9)	12	5.4 (1.9–15.3)
>1.5–3.0	15	3.6 (1.3–10.1)	14	3.8 (1.5–9.8)	9	5.2 (1.7–15.3)
>3.0–4.0	7	4.8 (1.5–15.5)	7	5.6 (1.9–16.5)	3	5.4 (1.3–22.4)
>4–5	4	3.9 (1.0–14.8)	5	5.2 (1.6–17.0)	1	3.6 (0.4–30.6)
>5–6	9	9.5 (3.1–29.3)	6	7.0 (2.2–22.0)	1	9.4 (1.1–81.6)
>6	47	16.3 (6.1–43.4)	46	18.8 (7.8–45.3)	2	6.0 (1.1–31.8)
Average exposure^‡^						
≤0.05	19	Reference				
>0.05–0.1	19	1.7 (0.9–3.4)				
>0.1–0.15	10	2.0 (0.9–4.5)				
>0.15–0.2	24	4.7 (2.4–9.4)				
>0.2	36	8.4 (4.5–15.8)				
Years employed						
≤10	5	Reference			5	Reference
>10–20	15	1.0 (0.4–2.8)			14	1.0 (0.4–2.7)
>20–30	23	1.4 (0.5–3.8)			16	1.2 (0.4–3.5)
>30	65	2.7 (1.0–7.3)			6	1.4 (0.4–4.8)
Smoking						
Never	25	Reference			13	Reference
Ever	49	2.2 (1.3–3.5)			24	1.8 (0.9–3.5)
Unknown	34	1.1 (0.6–1.8)			4	0.4 (0.1–1.2)

Also, HRs by smoking status controlling for age. ^‡^Additionally adjusted for duration of employment.

Although the majority (62%) of silicosis cases defined as ILO score 1/0 had individual cumulative exposure estimates greater than 3.0 mg/m^3^-years, 28 cases had cumulative exposures between 1.0 and 3.0 mg/m^3^-years, with relative risk estimates comparable to those with exposure up to 5.0 mg/m^3^-years, although HR estimates were sharply higher for exposures above that. However, among cohort members with estimated cumulative RCS exposures above 6.0 mg/m^3^-years, the HR was 16.3 (95% CI 6.1–43.4) based on 47 cases (43.5% of all 1/0 cases). Analyses lagging exposure by 10 years or limited to those hired since 1960 (again based on small numbers, especially in the higher exposure categories) were similar to the unlagged full cohort results, although the HR was elevated for the >0.5–1.0 mg/m^3^ cumulative exposure category (one exposure category lower than the ≥1/1 cases). Duration of exposure (in decades) was not a strong predictor of risk. Based on average exposure estimates, HRs clearly were elevated at levels greater than 0.15 mg/m^3^ (one exposure category higher than the ≥1/1 cases).

As with the ≥1/1 cases, smokers were at increased risk of silicosis defined as 1/0 but the association was weaker and not statistically significant. Analyses of silicosis cases defined as ILO score ≥ 1/0 by cumulative RCS exposure and stratified by smoking status generated somewhat attenuated associations for never and ever smokers while those with unknown smoking status were unchanged from the models adjusting for smoking (results not shown). Similarly, HRs were somewhat attenuated in models assuming those with unknown smoking status were smokers and non-smokers, respectively; however, risks still were increased among groups with cumulative RCS exposure >3.0 mg/m3-yrs (results not shown).

## Discussion

Although there are literally hundreds of papers on RCS and pulmonary diseases, there are relatively small numbers of epidemiological studies in which RCS specifically was quantitatively estimated and risks of silicosis or lung cancer were evaluated – and where some indicators of smoking status were available for each study member. The German Porcelain Workers Cohort Study is one of the largest to date evaluating cancer and silicosis risks among large groups of both men and women employed in porcelain manufacturing and followed over several decades, and where individual quantitative exposure estimates were derived from substantial historical industrial hygiene measurement data and detailed employment history records. According to the most recent systematic review, this study is one of only 10 published epidemiological studies considered of good quality and that quantitatively evaluated exposure-responses for crystalline silica and lung cancer, and one of three specifically addressing porcelain workers (the other two being the Chinese (11) and British (12, 13) pottery workers). This is one of eight such studies on silicosis: the only other that focused on pottery workers was the British study. This is one of only two studies included in the systematic review that addressed both silicosis and lung cancer.

The original publications on the German Porcelain Workers Cohort Study reflected a relatively short follow-up period (of about 20 years) with only 9.1% of the cohort deceased at the end of follow-up. In fact, a statistically significant deficit of lung cancer mortality was reported for men (SMR = 0.71, 95% CI 0.56–0.89) (6, 7). These characteristics specifically elicited criticisms regarding the scientific and regulatory value of the study results, especially in the context of the lack of any observed excess lung cancer mortality, a disease with an expected latency of about 20 or more years and high case fatality. For this reason, as well as to enhance the statistical power to detect excesses of other hypothesized cancers such as kidney cancer (of which only 9 and 5 deaths originally were observed among men and women, respectively) and renal disease (of which only 9 and 3 deaths originally were observed among men and women, respectively), we updated the mortality and silicosis incidence of the cohort through 2020, adding 15 years of follow-up. This resulted in roughly a threefold increase in the number of observed deaths including lung cancer deaths; a doubling of kidney cancer and renal disease deaths; but only a small increase in silicosis cases and deaths, likely due to the sharply reduced exposure to RCS over previous decades.

One particular strength of this study is the quantitative exposure estimation for each cohort member. Although gravimetric exposure sampling devices evolved over time, exposure chamber testing of vintage equipment was performed to derive 2,498 standardized exposure results for RCS between 1955 and 2006 to populate job exposure matrix cells [a detailed description of the exposure assessment is found in (9)]. This was made possible because all measurement data were centrally maintained at the Institute for Occupational Safety and Health (BGIA)—renamed in 2010 the Institute for Occupational Safety and Health of the German Social Accident Insurance (IFA)—of the German Statutory Accident Insurance (DGUV) agency in Sankt Augustin. All technological developments, work processes and exposure control measures were highly comparable across over 200 plants throughout Germany and over time. Other studies considered RCS exposures in the ceramics industry including porcelain workers, but we are aware of no other that exclusively focuses on the porcelain sector, a sector in Germany where RCS exposures were common and relatively well documented (and silicosis was known as “porcelain workers’ disease”).

Our decision not to update the exposure assessment was based on the uniformly negligible RCS exposure measurements obtained over the last several years. We were able to verify this in a feasibility study conducted in 2016–2017 that evaluated the most recent suite of crystalline silica industrial hygiene measurement data available for the porcelain industry. The median exposure level was determined to be 5 μg/m^3^ RCS, with little variation across the SEGs. This is consistent with or lower than the median concentration of 10 or < 10 μg/m^3^ measured across all SEGs in the last years of the original study follow-up, i.e., between 2000 and 2006 (9). Furthermore, many of the cohort members alive at the end of the original study retired over the extended follow-up, reducing or eliminating their potential for further exposure. Therefore, adding a trivial quantity of RCS to some workers due to continued negligible exposures over the update years unlikely would have impacted their cumulative exposure estimates or would have placed them in different exposure categories.

Another study strength was the ability to access some medical information (including smoking status and periodic chest radiographs of those working in areas where RCS exposure was likely) from the German occupational health surveillance program, which was nearly comprehensive for this cohort (6). This record also noted known RCS exposure from previous employment. Smoking information, although potentially available for each cohort member, was missing from health records for about 30% of the cohort. Unlike many studies evaluating occupational silica exposure and lung cancer risk with no individual-level smoking information, we were able to stratify by or adjust for smoking category using documented smoking status (ever vs. never for 70% of the cohort and “unknown” for the remainder). As expected, smoking was very strongly independently associated with lung cancer risk, with the HR = 17.9 for men and 6.1 for women. The statistically significantly increased lung cancer risk among those with unknown smoking status suggests that many of these workers might have been smokers. Analyses of silicosis risk by RCS category stratified by smoking showed that among those with unknown smoking status, HRs were lower among the three highest exposure groups than among the lowest two exposure groups (including the referent group), providing no evidence of a positive exposure-response relationship with silicosis exposure among this group. This lack of increased risk of silicosis among those with unknown smoking status suggests that their increased lung cancer risk was unlikely due to silica. Furthermore, re-run statistical models that assumed all workers with unknown smoking status were smokers, and that all were non-smokers, respectively, provided no indication of any relationship with RCS exposure. That the lack of association between RCS exposure at any level and increased lung cancer, regardless of smoking status, indicates that this finding unlikely resulted from or reflects confounding by smoking.

Possibly due to the declining rate of silicosis cases identified, the routine medical screening program was made voluntary for 2 years (2012–2013), and accordingly, the number of participants rapidly declined in that period. Nevertheless, we identified a small number (*n* = 8) of new cases based on radiographs obtained since the end of follow-up of the original study. These were evaluated according to the original study protocol. Additionally, a few cases were identified that had been reported to the statutory insurance program (i.e., Workers Compensation). We therefore could not identify additional silicosis cases among individuals that discontinued participation in the x-ray part of the health surveillance program, especially if they were asymptomatic or did not seek medical evaluation. However, because we ultimately were interested in evaluating the exposure thresholds at which silicosis risk clearly was increased (rather than quantify overall relative risk, as the association is already known to be causal), it likely is of little consequence that we slightly under-ascertained the total number of radiologically detectable silicosis cases.

Despite the large increase in numbers of deaths due to lung cancer for both men and women, and the additional 15 years of follow-up, we found no evidence of any excess of lung cancer deaths and no relationship with cumulative or average intensity exposures. The overall lack of an association was especially clear among men, where a 10% deficit was suggested. With a study size of approximately 18,000 and more than 300 expected lung cancer deaths (from the SMR analyses), the statistical power was over 99% to detect (at the alpha = 0.05 level) a relative risk for lung cancer of at least 1.5, and 80% power to detect a relative risk as small as 1.2. Furthermore, there was no support for any exposure-response by cumulative or average exposure categories and surrogates such as duration of employment, for which the HR was lower for both men and women in every group employed more than one decade (referent group). Given that the nearly 300 lung cancer deaths were reasonably distributed across cumulative exposure categories, Cox PH models were stable, and the resulting HRs were reasonably precisely estimated, reflected by the moderate to narrow estimated confidence intervals. However, due to the relatively small number of lung cancer deaths among non-smokers, we lacked sufficient statistical power to precisely estimate risks by exposure category among this group and subsequently to precisely statistically control for confounding by smoking.

To date, reported findings have been mixed across published studies estimating quantitative RCS exposure and evaluating the risk of lung cancer. Some, as with this updated study, reported no clear increased risk associated with silica exposure [e.g., (3, 12, 14, 15)]. Others reported statistically significant relative risks (5, 11, 16–18) or increased RR estimates for lung cancer but without reporting confidence intervals (19). Clearly increased relative risk estimates for lung cancer typically were reported among groups with relatively or very high cumulative exposures, although Wang et al. (11) and Bugge et al. (20) reported increased relative risks at nearly all exposure levels. Reported cumulative exposure levels at which relative risks were increased were, e.g., ≥2.4 mg/m^3^-years (5); >5.6 mg/m^3^-years (16); >6.3 mg/m^3^-years (17); and > 10 mg/m^3^-years (18). Although most of these apparent exposure threshold estimates were derived from statistical analyses controlling for smoking, the Ge et al. (5) result is different in that it represents a subgroup of never smokers, greatly eliminating the potential for residual confounding that likely was present in their other analyses.

The strong and statistically significant relationship between RCS exposure and x-ray verified silicosis morbidity indicates that the lack of any association with lung cancer in our study unlikely was due to an invalid exposure assessment method and reinforces the well-established causal relationship between RCS exposure and silicosis risk. Furthermore, because RCS-exposed workers received chest radiographs about triennially, there likely was a greater opportunity to identify early phase and asymptomatic lung tumors, which would tend to increase the numbers observed. Nevertheless, there was no evidence of any detection bias. More interesting, however, are questions related to the amount of RCS exposure that increases silicosis risk, i.e., exposure thresholds. Because we identified eight new silicosis cases (defined as ILO score ≥ 1/1) over the 15-year extended follow-up, only a 20% increase, our findings of exposure thresholds >3 mg/m^3^-years cumulative and > 0.10 mg/m^3^ were unchanged from the original study.

When we defined silicosis as ILO grade 1/0, however, the number of cases was much larger, with 49 (47 in men and 2 in women) of the total 108 falling in the cumulative exposure category of >6 mg/m^3^-years, with an estimated HR for men of 16.3 (95% CI 6.1–43.4). The HR was relatively substantially increased for all groups starting with those in the >1.0–1.5 mg/m^3^-years exposure category, in contrast with those defined as ILO score ≥ 1/1, where the risk was not substantially increased until cumulative exposure exceeded 3 or 4 mg/m^3^-years. Most other published studies examining quantitative level of cumulative RCS exposure and silicosis (generally including all cases scored as ≥1/0) demonstrated clearly increased relative risks at higher exposures. However, one study (10) reported increased RR estimates (ranging from 1.86 to 3.90, respectively) in all industrial groups, including tungsten miners, iron and copper miners, tin miners and pottery factory workers. The difference we observed in apparent cumulative exposure thresholds between silicosis cases defined as 1/0 versus ≥1/1 may indicate that the 1/0 classification level is a more sensitive, but also less certain, i.e., less specific indicator of increased silicosis risk. It also may be due either to exposure misclassification, especially those considered least exposed (as there are no true silicosis cases that are unexposed), or to imprecision, given the relatively small number of cases in each exposure category. On the other hand, our analysis of the silicosis cases defined as ILO category 1/0 suggested an exposure intensity threshold that was slightly higher than that observed for ILO category ≥1/1 cases, i.e., >0.15 mg/m^3^ vs. >0.10 mg/m^3^.

Our reported finding of increased silicosis (defined as ≥1/1) risk among known smokers is difficult to understand, as smoking is not believed to be a risk factor for silicosis. A recent cohort study focused on risk factors for silicosis progression noted that their study was possibly the first to examine possible risk factors for progression. They indicated that the type of RCS exposure (manufactured vs. natural stone) and type of silicosis (i.e., complicated vs. simple) were associated with silicosis progression, but not age at diagnosis, baseline pulmonary function or smoking (21). One possible explanation for the increased risk seen among smokers in this study might be that due to their smoking (especially on the job), smokers receive a greater inhaled dose of RCS form dust on their hands and cigarettes than those not actively bringing silica-contaminated hands into their breathing zones. However, if this were true in this study, it also might have been reported in other studies, but we are unaware of such reports. Smoking also might cause changes in chest radiographs suggesting silicosis, leading to more false positive classifications; however, the observed relationship was much stronger among silicosis ≥1/1 cases which would be expected to have greater diagnostic certainty, than the 1/0 cases. Another hypothesis is that the association arises from the correlation between smoking and RCS exposure occurring in earlier decades when silica exposures were the highest and smoking was more prevalent. Some supportive evidence of this was seen in analyses of silicosis risk by RCS exposure category, stratified by smoking status: the largest HR estimate was that for those of unknown smoking status in the highest RCS category (i.e., >6.0 mg/m^3^-yrs.), and that single subgroup also had the largest number of silicosis cases.

This 15-year updated cohort study largely confirmed the results of the original study demonstrating no increased risk of lung cancer in the German Porcelain Workers Cohort Study. Also consistent with the original findings, this update demonstrated strong associations between cumulative and average RCS estimates and risk of silicosis for both ILO category 1/0 and ≥ 1/1 cases, with clear indications of exposure thresholds that can be further quantified.

## Data Availability

The datasets presented in this article are not readily available because they are owned by the German Statutory Accidence Insurance Association of the BG Administrative Sector (VBG). Requests to access the datasets should be directed to kmundt@umass.edu.
