# Supplementary material for: Results of 15 years of extended follow-up of the German porcelain workers cohort study: lung cancer and silicosis
Source: Front Public Health. 2025 Mar 18;13:1552687. doi: 10.3389/fpubh.2025.1552687 (PMC11959091; doi:10.3389/fpubh.2025.1552687)
Supplement: Supplementary file 1 [file Table_1.DOCX]

Supplemental Tables

**Supplemental Table 1**. **SMR and 95% CIs for Selected Categories of Cause of Death among Men (Full Cohort)**

| **Cause of Death** | **ICD-10** | **Observed** | **Expected** | **SMR** | **Lower 95% CI** | **Upper 95% CI** |
| --- | --- | --- | --- | --- | --- | --- |
| All causes | A00–Y98 | 2808 | 2559 | 1.10 | 1.06 | 1.14 |
| Infective and parasitic diseases | A00–B99 | 57 | 44 | 1.29 | 0.99 | 1.67 |
| Malignant neoplasms | C00–C97 | 873 | 800 | 1.09 | 1.02 | 1.17 |
| Buccal cavity and pharynx | C00–C14 | 48 | 34 | 1.42 | 1.07 | 1.88 |
| Esophagus | C15 | 44 | 30 | 1.46 | 1.09 | 1.96 |
| Stomach | C16 | 36 | 41 | 0.88 | 0.63 | 1.22 |
| Large intestine | C18 | 74 | 57 | 1.31 | 1.04 | 1.64 |
| Liver and gallbladder | C22–C25 | 120 | 89 | 1.35 | 1.13 | 1.62 |
| Pancreatic cancer | C25 | 67 | 51 | 1.32 | 1.04 | 1.67 |
| Lung | C34 | 194 | 215 | 0.90 | 0.78 | 1.04 |
| Breast | C50 | 4 | 1 | 3.39 | 0.92 | 8.68 |
| Prostate | C61 | 60 | 66 | 0.91 | 0.71 | 1.18 |
| Bladder | C67 | 18 | 21 | 0.85 | 0.54 | 1.36 |
| Kidney | C64 | 23 | 23 | 1.01 | 0.67 | 1.52 |
| Leukemia | C91–C95 | 20 | 26 | 0.78 | 0.50 | 1.20 |
| Diabetes | E10 –E14 | 64 | 55 | 1.16 | 0.91 | 1.48 |
| Mental disorders | F00–F99 | 54 | 77 | 0.70 | 0.54 | 0.91 |
| Nervous system and sensory organs | G00–G99, H00 –H95 | 48 | 64 | 0.75 | 0.56 | 0.99 |
| Diseases of the circulatory system | I00–I99 | 925 | 862 | 1.07 | 1.01 | 1.14 |
| Diseases of the respiratory system | J00–J98 | 178 | 167 | 1.06 | 0.92 | 1.23 |
| Diseases of the digestive system | K00–K93 | 169 | 154 | 1.10 | 0.94 | 1.28 |
| Cirrhosis | K70, K73–K74, K76.0 | 86 | 90 | 0.95 | 0.77 | 1.18 |
| Diseases of the genitourinary system | N00–N09 | 24 | 40 | 0.60 | 0.40 | 0.90 |
| Renal disease | N00–N08, N10 –N12, N14 –N19, N26 –N29 | 22 | 31 | 0.71 | 0.47 | 1.08 |
| Ill– defined conditions | R00–R99 | 38 | 88 | 0.43 | 0.31 | 0.59 |
| External causes | V01–Y98 | 106 | 158 | 0.67 | 0.55 | 0.81 |

**Supplemental Table 2. SMR and 95% CIs for Selected Categories of Cause of Death for Women (Full Cohort)**

| **Cause of Death** | **ICD-10** | **Observed** | **Expected** | **SMR** | **Lower 95% CI** | **Upper 95% CI** |
| --- | --- | --- | --- | --- | --- | --- |
| All causes | A00–Y98 | 1778 | 1906 | 0.93 | 0.89 | 0.98 |
| Infective and parasitic diseases | A00–B99 | 49 | 35 | 1.40 | 1.06 | 1.85 |
| Malignant neoplasms | C00–C97 | 557 | 640 | 0.87 | 0.80 | 0.95 |
| Buccal cavity and pharynx | C00–C14 | 14 | 9 | 1.58 | 0.93 | 2.66 |
| Esophagus | C15 | 2 | 7 | 0.27 | 0.03 | 0.98 |
| Stomach | C16 | 25 | 26 | 0.96 | 0.65 | 1.42 |
| Large intestine | C18 | 43 | 47 | 0.92 | 0.69 | 1.25 |
| Liver and gallbladder | C22–C25 | 66 | 70 | 0.94 | 0.74 | 1.20 |
| Pancreatic cancer | C25 | 35 | 46 | 0.76 | 0.55 | 1.06 |
| Lung | C34 | 90 | 92 | 0.98 | 0.80 | 1.21 |
| Breast | C50 | 100 | 132 | 0.76 | 0.62 | 0.92 |
| Kidney | C64 | 14 | 13 | 1.11 | 0.66 | 1.88 |
| Leukemia | C91–C95 | 17 | 20 | 0.84 | 0.52 | 1.36 |
| Diabetes | E10 –E14 | 55 | 48 | 1.14 | 0.88 | 1.49 |
| Mental disorders | F00–F99 | 41 | 72 | 0.57 | 0.42 | 0.78 |
| Nervous system and sensory organs | G00–G99, H00 –H95 | 39 | 58 | 0.67 | 0.49 | 0.92 |
| Diseases of the circulatory system | I00–I99 | 607 | 617 | 0.98 | 0.91 | 1.07 |
| Diseases of the respiratory system | J00–J98 | 114 | 110 | 1.03 | 0.86 | 1.24 |
| Diseases of the digestive system | K00–K93 | 103 | 100 | 1.03 | 0.85 | 1.25 |
| Cirrhosis | K70, K73–K74, K76.0 | 45 | 49 | 0.92 | 0.68 | 1.23 |
| Diseases of the genitourinary system | N00–N09 | 24 | 39 | 0.61 | 0.41 | 0.92 |
| Renal disease | N00–N08, N10 –N12, N14 –N19, N26 –N29 | 18 | 30 | 0.61 | 0.38 | 0.97 |
| Ill– defined conditions | R00–R99 | 26 | 52 | 0.50 | 0.34 | 0.73 |
| External causes | V01–Y98 | 29 | 79 | 0.37 | 0.26 | 0.53 |

**Supplemental Table 3. Characteristics of Cohort Members with Chest Radiograph ILO score 1/0; ILO score ≥1/1 and Negative or No reading**

|  | ILO score 1/0  (n=108) | | ILO score ≥1/1  (n=48) | | Negative or no reading  (n=17,424) | |
| --- | --- | --- | --- | --- | --- | --- |
|  | n | % | n | % | n | % |
| Sex |  |  |  |  |  |  |
| Female | 28 | 25.9 | 7 | 14.6 | 9,280 | 53.3 |
| Male | 80 | 74.1 | 41 | 85.4 | 8,144 | 46.7 |
|  |  |  |  |  |  |  |
| Decade of hire |  |  |  |  |  |  |
| <1950 | 20 | 18.5 | 11 | 22.9 | 697 | 4.0 |
| 1950-1959 | 47 | 43.5 | 17 | 35.4 | 2,778 | 15.9 |
| 1960-1969 | 22 | 20.4 | 8 | 16.7 | 2,561 | 14.7 |
| 1970-1979 | 16 | 14.8 | 7 | 14.6 | 4,900 | 28.1 |
| ≥1980 | 3 | 2.8 | 5 | 10.4 | 6,488 | 37.3 |
|  |  |  |  |  |  |  |
| Years employed |  |  |  |  |  |  |
| ≤10 | 5 | 4.6 | 3 | 6.2 | 4,594 | 26.3 |
| >10-20 | 15 | 13.9 | 6 | 12.5 | 4,735 | 27.2 |
| >20-30 | 23 | 21.3 | 15 | 31.3 | 4,125 | 23.7 |
| >30 | 65 | 60.2 | 24 | 50.0 | 3,970 | 22.8 |
|  |  |  |  |  |  |  |
| Smoking status |  |  |  |  |  |  |
| Ever | 49 | 45.4 | 24 | 50.0 | 6,542 | 37.5 |
| Never | 25 | 23.1 | 12 | 25.0 | 5,401 | 31.0 |
| Unknown | 34 | 31.5 | 12 | 25.0 | 5,481 | 31.5 |
|  |  |  |  |  |  |  |
| Prior silica exposure |  |  |  |  |  |  |
| Probable | 9 | 8.3 | 9 | 18.7 | 652 | 3.7 |
| Possible | 13 | 12.1 | 7 | 14.6 | 1,445 | 8.3 |
| Unlikely | 82 | 75.9 | 30 | 62.5 | 14,252 | 81.8 |
| Unknown | 4 | 3.7 | 2 | 4.2 | 1,075 | 6.2 |
